# Preceptorship of Student Nurses in Ghana: A Descriptive Phenomenology Study

**DOI:** 10.1155/2021/8844431

**Published:** 2021-01-08

**Authors:** Nancy Innocentia Ebu Enyan, Christian Makafui Boso, Sarah Ama Amoo

**Affiliations:** ^1^Department of Adult Health, School of Nursing and Midwifery, University of Cape Coast, Cape Coast, Ghana; ^2^Cape Coast Teaching Hospital, Cape Coast, Ghana

## Abstract

**Background:**

Preceptorship plays an integral part in the clinical training of nursing and midwifery students, especially in high-income countries where it is a well-accepted concept. However, in Ghana, most nurses and midwives do not view preceptorship as part of their role.

**Aim:**

The aim of this study was to explore the lived experiences of preceptorship of student nurses and the challenges confronting the preceptorship role.

**Methods:**

A descriptive phenomenological study was conducted with 22 purposively selected preceptors aged 34 to 56 years from five clinical placement sites in the Cape Coast Metropolis in the Central Region of Ghana. Most of the participants had been preceptors for two to 18 years. In-depth interviews were conducted with the aid of a semistructured interview guide and analysed by qualitative thematic analysis inspired by Braun and Clarke's description of the method.

**Results:**

The essence of the phenomenon has been captured in three main themes: (1) being excited about the role as it offered opportunities to learn and build relationship with students. (2) Encountering challenges including student's unwillingness to learn, absenteeism, and disrespect and also lack of interest of staff to assist students, time constraints, workload, burnout, parallel schedules of preceptors, and large student numbers, and (3) the need for effective collaboration between educational institutions and hospitals.

**Conclusions:**

Though preceptors were excited about precepting student nurses, the challenges associated with it are multidimensional which requires effective collaboration between educational institutions and clinical placement sites.

## 1. Background

Preceptorship constitutes an integral part in the clinical training of nursing and midwifery students, especially in high-income countries where it is a well-accepted concept. The process of preceptorship is conceptualized as a developmental relationship in which an experienced and knowledgeable individual assists the less experienced or novice person to acquire certain competencies through constructive guidance and support [1]. Preceptorship in this context refers to a registered nurse supporting student learning in practice. Clinical preceptorship enhances the development of shared responsibility for education, training, and increased job satisfaction [2]. Several studies conducted in developed economies have highlighted the effectiveness of preceptorship in improving a competency-based profession like nursing [3–5]. According to Horton et al. [5], it is imperative that preceptors are well trained and resourced to function effectively in the preceptorship position. These, they believe, will empower the preceptors to deliver on their mandate. The lack of guidelines defining professional responsibility as a preceptor and support for preceptors with resources, information, and recognition affects their ability to effectively work in the preceptor role [6]. Madhavanpraphakaran et al. [7] and Paton [8] added that preceptors need to be acknowledged, supported, and guided in performing their unique professional teaching practice, which is different from their role as clinically competent professional nurses. In supporting preceptors to effectively manage their roles, the cognitive learning theory emphasizes experience as a critical factor in learning and development. Therefore, learners construct knowledge based on their experience and social interaction [9].

Precepting has been adopted by the nursing and midwifery professions as a critical intervention to prepare nursing and midwifery students for practice to effectively cope with various roles in nursing, midwifery, and the environment in which they practice [10]. Therefore, attempts to precept and instill the culture of nursing including caring, commitment, critical thinking, and compassion into these novice nurses should be the responsibility of every practicing nurse. Additionally, sustainable development goal four focuses on quality education which suggests the need for innovative measures to enhance nursing and midwifery education in Ghana [11].

In Ghana, it is assumed that most nurses and midwives do not view precepting as part of their role. Rather, it is perceived as an extra burden or responsibility which requires some rewards. Nursing and midwifery students are the future of the nursing and midwifery professions and efforts to inculcate the culture of the profession and socialize them to develop right attitudes are necessary in enhancing their skills and attitudes and to shape the image of the profession. A narrative review conducted by Atakro and Gross [12] identified inadequately prepared preceptors, lack of qualified nurses, and midwives for the preceptor role and inadequate supervision from nursing faculty as the main challenges faced by preceptors. However, the studies involved in the review were mainly conducted in advanced settings and their applicability to the Ghanaian context may be unclear. Again, a previous study using the ethnographic approach to describe the experiences of nurse educators, students, and preceptors in Ghana reported that the clinical teaching strategy being used was not consistent with the concept of preceptorship [13]. In Ghana, many nursing schools that run the 4-year bachelor's degree in nursing programme introduce their students to clinical nursing practice in their second year, while students enrolled in the 3-year diploma in nursing programmes start undertaking clinical nursing in the first year. Though clinical nursing practice in hospitals requires the assistance of preceptors, there has not been a well organised preceptorship programme to enable both faculty and preceptors to effectively collaborate in training students [13]. The current practice in Ghana is that every nurse is assumed to be a preceptor, therefore when undergraduate students are assigned to the wards, they report to the ward in-charges/managers, who either supervise these students themselves or assign them to other nurses to supervise their activities in the ward. Some schools have identified and partnered with some experienced nurses to precept their students during clinical practice. In their absence, students are left on their own with no proper supervision. In some African settings including South Africa and Botswana, there are well established preceptorship programmes [14, 15], where final year students closely work with preceptors to achieve the objectives of clinical placement [14]. This is not the case in Ghana; with no proper training and a clear definition of what their responsibility towards students is, some preceptors are ill prepared to carry out the preceptor role. This study, therefore, sought to explore preceptors' understanding of preceptorship of student nurses and the challenges confronting the preceptorship role in the Cape Coast Metropolis of Ghana.

## 2. Methods

A descriptive phenomenology study was conducted in the Cape Coast Metropolis in the Central Region of Ghana to explore the lived experiences of preceptors. Phenomenology aims at describing a specific phenomenon as lived experience [16]. Lived experience provides a meaning to how an individual perceives a particular phenomenon, presenting the reality of the experience in the individual's life [17]. Phenomenological analysis seeks to clarify the essence of the phenomena [17]. Preceptors go through varied experiences as they closely work with nursing students. Although precepting nursing students can be rewarding, it is not without challenges [18]. The descriptive phenomenological approach was suitable in exploring the subjective experience of preceptors. Additionally, there is paucity of research on the lived experience of preceptors within the Ghanaian context and, as a result, the need for research exploring the lived experience in order to clearly understand the phenomenon. Professional nurses and midwives who have ever precepted within the Cape Coast Metropolis constituted the population for this inquiry. The selection of this design helped in exploring the phenomenon of preceptorship as it has not been rigorously researched within the Ghanaian context. Therefore, the design facilitated a deeper understanding of the concept of preceptorship from the perspectives of these preceptors.

With the help of the nurse managers, participants were drawn from five health facilities that offered clinical placements for nursing and midwifery students in the Cape Coast Metropolis. The maximum variation technique of purposive sampling was adopted to select preceptors who have ever precepted a nursing student before registration with the Nursing and Midwifery Council of Ghana. A semistructured interview guide was developed by the first and third authors to facilitate the data collection. The guide covered the following questions: which part of being a preceptor do you find enjoyable or rewarding? What challenges confront the preceptorship role? Based on your experience, how do you think the preceptor's role can be improved? These were followed by probing questions to solicit thorough information from the participants. The questions were based on gaps in knowledge found by reviewing the literature. The demographic information collected from participants included their age, gender, educational level, number of years work experience, and number of years having served as a preceptor. The semistructured interview guide was pretested on four nurse preceptors in a facility that did not form part of this study, responses participants gave indicated that the questions asked were understood, therefore the interview guide was not altered. The participants who met the criteria in the varied health facilities were identified and interviewed.

The first and third authors interviewed participants face to face. Arrangements were made to meet the selected participants at work and they were interviewed at their nurses room in the ward after they had closed from work. The interviews were in-depth and lasted between 30 to 45 minutes for each participant. Field notes were taken and interviews were audio recorded with permission from the participants. The data were transcribed after each interview session. The members of the research team reviewed the interviews for richness of information and variations. When no new data were emerging from the interviews, it was stopped after interviewing the 22^nd^ participant. Those who participated were aged 34 to 56 years with at least two years of experience in preceptorship.

Data collection and initial analysis were done simultaneously to examine the data for new information. The data were transcribed verbatim by the second and third authors and the transcript cross-checked with the tapes to determine the accuracy of the information. The data analysis followed Braun and Clarke's [19] procedures for thematic analysis. Sundler et al. [20] affirmed the use of qualitative thematic analysis in descriptive phenomenology. Before coding the data manually, we immersed ourselves in the data by reading through transcripts repeatedly to familiarise ourselves with the data. This process helped shape ideas about the possible meanings and patterns in the data. The data were thoroughly coded by using numbers and letters to represent the participants and efforts were made to ensure inclusiveness of all the data. Also, individual extracts from the data were coded into the most appropriate themes. After identifying the different codes across the dataset, the many different codes were examined and sorted to form a broader theme. The identified themes were reviewed against the organised extract for coherence in the pattern.

A reflexive journal was maintained and rigor attained by ensuring that credibility, transferability, dependability, and confirmability were strictly adhered to [21]. Transferability was ensured through description of the setting and characteristics of the sample. Techniques to achieve dependability and confirmability include verbatim exemplary quotations to support the results and detail description of the methodology. The second and third authors separately analysed the data and conferred to agree on the themes. Differences were resolved through discussion. The researchers engaged with the preceptors in a manner that facilitated thick and rich description of the phenomenon of preceptorship which could lead to sound conclusions. Member checking was employed by sharing the preliminary findings with the participants, asking them for feedback and incorporating their feedback into the conclusions drawn. The researchers engaged in peer debriefing by discussing study with an expert to provide a thoughtful critique about the methodology and subject content. Furthermore, procedures instituted to ensure that the findings were dependable include the use of an inquiry audit as proposed by Guba and Lincoln [21]. Consequently, an audit trail was maintained by keeping records of the field notes, tape recordings, data analysis products, coding schemes created, coded data, themes emerged, and interpretations made. The findings of this study will be applicable to clinical placement sites within the Cape Coast Metropolis of Ghana. It could also be extrapolated in similar clinical settings across the region.

## 3. Results

Twenty-two preceptors participated which consisted of six males and 16 females with an age range of 34 to 56 years. Of the 22 participants, eight held graduate/postgraduate and 14 held undergraduate degrees. The participants had been working as nursing professionals for 10 to 21 years. Eighteen of the participants had been preceptors for two to 18 years. However, four of them could not recollect when they became preceptors, as presented in Table 1.

Three main themes and several subthemes emerged that reflected the lived experiences of the preceptors (Table 2). Theme one reflected participants being excited by the preceptorship role; theme two described challenges confronting the preceptorship; and theme three described the views of participants on ways of improving preceptorship based on their experience.

### 3.1. Being Excited by the Preceptorship Role

Nursing is a practice-oriented profession and nursing students require assistance in the clinical setting to help them link theory to practice; this is where the role of the preceptor becomes invaluable. This theme relates to the factors that motivate participants to partake in the preceptorship programme. According to the participants, they were motivated by the opportunity to continuously read/study and teach, the opportunity to build a relationship with students, and acknowledgement or recognition they receive from students. Other participants were motivated when they see the students' progress to become competent professionals. These motivational factors are indicated in the following quotes:

“The reading and then getting the students around and teaching them are something that I like (P1).”

“Seeing my students doing well, coming out successfully as professional nurses and…they also putting up their best in their work give me that joy to keep on training them to assist in the work that we are doing (P3).”

*“It's about relationship with students. Anytime you see them, you walk around the point at you and say “this is sir moving.” Sometimes they give you fans. That is the most exciting aspect of it (P2)*.”

*“Sometimes when I meet people that have gone through my hands, the respect they give me and the fact that I see that they are also good and I go somewhere and they say this guy is a very good nurse... Or I enter a particular facility, nurses come to me and say “…thank you for what you have done for me,” I feel satisfied with that. I think money is not more than that personal acknowledgement that you have contributed to my success, it's good for me. There are times you go, you are looking for certain things and your students are available to just help you to do it and within no time you are out of the facility it's good (P17)*.”

### 3.2. Challenges Confronting Preceptorship

Participants of this study identified a number of challenges confronting preceptorship which denotes three main subthemes. These challenges were students' factors, preceptors' factors, and institutional factors. The participants' highlighted students' unwillingness to learn. This is manifested in students' absenteeism/truancy and idling and students' not obeying instructions/disrespect. The participants also narrated some difficulties relating to preceptors' that have significantly impacted the preceptorship role which included lack of interest to help students, time constraint, increased workload, burnout, and parallel schedules. Other institutional level factors reported by the participants included large student numbers, lack of logistics/equipment (teaching materials/aids), lack of collaboration between academic institutions and preceptors, lack of remuneration, and lack of training specific to the preceptor role.

The following excerpts support this theme:

“Some of the students are not ready to learn…Another challenge is that…equipment to work with is sometimes a challenge. You need this, it's not available…so, you need to almost always improvise…(P1).”

*“Some of the challenges are students not obeying instructions given to them; students not coming to work as they are supposed to and also being…disrespectful... When you give them instructions and they refuse to go according to the instruction given to them, it gives you a lot of work to do. Sometimes you assign them and they run away (P3)*.*”*

*“…the number [students] is large, it becomes difficult to be able to attend to every student, and within the time limit that we work, we are not able to (attend to students) because some come in the afternoon. So, supposing I work in the morning, there should be somebody in the afternoon... If we have more preceptors, I think it will help (P5)*.*”*

*“...It is very very stressful…Some (students) are not really ready…I have forty students in the facility… Sometimes it becomes…just a few hands… (helping students) … You are not given anything (P1 2)*.*”*

### 3.3. Improving Preceptorship

The participants believed that preceptorship as practiced today needed to be improved if the full benefits of the preceptorship model are to be derived. The measures they suggested included effective collaboration between educational institutions and the hospitals, improved interdisciplinary approach to teaching, financial reward to preceptors, and the need to streamline the preceptorship role. Specific suggestions for effective collaboration were improved communication between schools and hospitals, preceptors' accountability for their stewardship through periodic reports to the schools, regular meetings of stakeholders, and preparation for the preceptor role. They also suggested that schools should select their own preceptors. These suggestions are captured in the following quotes:

*“The schools should link up with the hospitals; communicate with the DNS, the nurse in-charge as well as some of the nurses on the ward (P1)*.*”*

*“Organizing meetings, getting the feedback to the individuals, and knowing what must be done. Then, remuneration depending on the institution's ability (P4)*.”

*The training institutions need to formally communicate to the people they want to be preceptors. Then, they may have to train. Let the people know what is expected of them as preceptors, and then there should be that involvement where they are made to feel part of the training school not as in going to teach but then they have an input to make when it comes to the clinical aspect (P9)*.”

## 4. Discussion

### 4.1. Being Excited about the Preceptorship Role

Preceptorship is highly beneficial to nursing as it facilitates the acquisition of skills and has the potential of transforming the profession. Strong preceptorship is also necessary for a practice profession as it helps bridge the theory-practice gap [22–24]. The findings revealed aspects of the preceptorship that the preceptors found enjoyable or rewarding to continuously function in that role. It was evident that preceptorship offered opportunities for the preceptors to learn, build relationship with students, and facilitated the progression of students from novice to competent professionals. An appreciative inquiry conducted highlighted the desire for reciprocal learning and friendship as some of the intrinsic benefits to functioning in the preceptor role [25]. Furthermore, preceptors' coach and guide nursing students thereby helping them acquire certain clinical competencies to gradually become proficient in their roles [26]. The role can be viewed as rewarding providing opportunities for inexperienced nurses to learn and build competence [27].

Participants reported that acknowledgement and recognition by students were additional sources of motivation. It is interesting to note that a similar finding was reported by Asirifi et al. [13] as the preceptors were interested in being recognised in the form of receiving a special pin that will distinguish them from those not functioning in that role. Latfrance [25] emphasised acknowledgement as one of the key factors that drive preceptors in performing their roles. The preceptor role is inherently satisfying [28]. However, the factors that motivated preceptors highlighted in this study are as well found in other studies which include incentive to teach [23] and facilitating the development of the novice nurses to competent professionals [23]. The background characteristics of the participants may be similar across the studies which might have accounted for the observed findings. Therefore, efforts to improve preceptorship of student nurses need to consider these factors to maximize the experiences of preceptors.

### 4.2. Challenges Confronting Preceptorship

The challenges with the preceptorship programme found in this study were multifaceted with some related to students and preceptors while others emanated from the educational institutions. Student-related factors including the unwillingness to learn, disobeying instructions, disrespect, absenteeism, and idling during clinical placement are a major worry to their professional development. A previous study reported the need to recognize and manage students who demonstrate inappropriate behaviours, supporting them to come out of those untoward behaviours and introducing evaluation systems to foster success at the clinical area [29]. This requires that effective collaboration between faculty and preceptors is necessary in instituting clinical policies to manage untoward behaviours at the clinical settings [18, 30]. Additionally, effective supervision of students on placement will deter them from exhibiting inappropriate behaviours as empirical evidence from a cross-sectional study conducted in South Africa involving preceptors and preceptees, and unit managers suggest that some nurses fail to support students during clinical placement [31]. This finding may be applicable to the Ghanaian context as similar attitudes have been observed in the clinical setting.

Challenges specific to the preceptors and educational institutions about the preceptorship programme reported in this study are consistent with other studies [27, 32]. These include lack of interest to assist students, time constraints, increased workload, burnout, parallel schedules, and lack of equipment. This suggests that problems preceptors encounter with nursing students are similar across different settings [18, 30, 33–35]. A previous study conducted in a high-income setting reported a feeling of unpreparedness as one of the reasons nurses do not want to participate in preceptorship [32]. Other challenges consistent with other studies included personality clashes, time constraint/increased workload and lack of motivation of students, lack of organizational support/collaboration, and student placement coinciding with preceptors' clinical duties [18, 30, 33–35]. An earlier study conducted among midwives in four African countries reported increased workload as a major factor impacting preceptorship [36]. Additionally, large students' numbers, lack of logistics/equipment, of remuneration and of training specific for preceptors emerged from this study which educational institutions need to critically examine to curtail the challenges and enhance preceptorship. Even, in an advanced setting, logistical elements were noted to have impacted precepting of students at the clinical sites [37].

### 4.3. Improving Preceptorship

The findings suggest that effective collaboration between educational institutions and healthcare agencies, improving interdisciplinary approach to teaching, financial reward for preceptors, preparation for the role, and streamline preceptorship are some of the strategies for improving preceptorship. The measures highlighted by participants necessary for improving preceptorship have been noted in the nursing literature. For instance, in the area of collaboration, improved communication, preceptors being accountable for their stewardship, regular meetings, and schools selecting their own preceptors emerged. These findings affirm a previous study conducted by Asirifi et al. [13] in Ghana. Effective collaboration between nursing educational institutions, healthcare agencies, and preceptors are critical in helping students achieve their learning outcomes and the overall educational goals. This calls for a redefining of the concept of preceptorship between stakeholders in the educational process to agree on a contextually relevant preceptorship model that can maximize students' learning [18]. A well-developed preceptorship model within the Ghanaian context will streamline its implementation in varied nursing educational institutions. Additionally, interdisciplinary approach to teaching also emerged. This requires nursing faculty to develop collaborative clinical teaching models that can offer nursing students the opportunity to present joint clinical case conferences with their interprofessional peers [38]. The collaborative process allows students to appreciate the expertise of other disciplines and prepare them for future clinical partnerships.

It is worth mentioning that preceptors can effectively engage with students if they are well prepared for the role. It is plausible to assume that some nurses' and midwives' function in the preceptor role without receiving any formal training or participate in continuing professional development programmes on preceptorship which can affect their ability to meet the demands of the role.

Therefore, there is the need for preceptors to receive educational preparation toward the preceptor role [39–41]. According to Burns et al. [40], developing individuals for the preceptor role, is beneficial to the student, preceptor, and faculty with more effective and less stressful clinical teaching. Also, the benefit of preceptors being accountable through periodic evaluation reports to the academic institutions and students identified in this study has been noted [23]. The role of the preceptor will benefit from formal educational preparation to better assist students in acquiring the necessary clinical skills and knowledge for effective nursing care [41].

Financial rewards for preceptors emerged as a strategy for improving preceptorship which has been cited by a previous study [18]. A possible explanation of this finding is that in Ghana most nurses and midwives perform the preceptorship role in addition to their regular clinical schedules at the varied hospitals. Some institutions renumerate them based on the agreed number of clinical hours they spend with the students. However, other training institutions may not have systems in place to recognize the efforts of the preceptors. Meanwhile, organizing free workshops or continuing professional development programmes for preceptors and issuing them with certificates to enable them renew their professional identification numbers can be a great source of motivation [18].

## 5. Conclusions

Preceptorship is central to enabling students to relate theory to practice. Preceptors play significant roles in students' acquisition of knowledge and competencies to transit from the role of student to that of a registered nurse or midwife. Though preceptors find their role as exciting, there are complex inherent challenges in the preceptorship role that educational institutions and healthcare facilities/agencies need to address to foster precepting, enhance the process, and improve the clinical learning experiences of students. This study highlights the preceptors' experiences of preceptorship and the challenges confronting preceptorship in a low-middle-income country.

## Figures and Tables

**Table 1:** Description of participants' characteristics.

Participants	Age	Gender	Work experience	Years of preceptorship	Educational level
1	34	F	10	4	Master of nursing
2	45	M	15	6	Master of nursing
3	41	F	17	6	BSc. nursing
4	56	M	19	Cannot remember	BSc. nursing
5	37	F	12	2	BSc. nursing
6	34	F	10	Cannot remember	MSc. nursing
7	41	F	17	10	Master of nursing
8	37	M	13	10	BSc. nursing
9	34	F	12	7	BSc. nursing
10	42	F	16	3	BSc. nursing
11	38	F	13	11	MSc. nursing
12	42	F	15	14	BSc. nursing
13	49	F	18	Can't remember	BSc. nursing
14	36	F	12	9	BSc. nursing
15	46	F	21	Can't remember	BSc. nursing
16	42	F	16	6	MSc. nursing
17	43	M	20	10	MSc. nursing
18	48	M	17	4	MSc. nursing
19	34	M	11	8	BSc. nursing
20	37	F	14	4	BSc. nursing
21	50	M	20	10	BSc. nursing
22	46	F	20	18	BSc. nursing

**Table 2:** Thematic table.

Main themes	Subthemes: code
Preceptorship being exciting or rewarding	Personal motivation
	(i) Learn
	(ii) Build relationship with students
	(iii) Novice to competent professionals
	Acknowledgement and recognition by students

Challenges confronting the preceptorship	Students' factors
	(i) Unwillingness to learn
	(ii) Students not obeying instructions/disrespect
	(iii) Absenteeism/truancy, idling
	(iv) Parallel schedules of students
	Preceptors' factors
	(i) Lack of interest of staff to help students
	(ii) Time constraint/workload
	(iii) Burnout of preceptors
	(iv) Parallel schedules of preceptors
	Institutional level factors
	(i) Large student numbers
	(ii) Lack of logistics/equipment/teaching materials/aids
	(iii) Lack of collaboration between school and preceptors
	(iv) Lack of remuneration
	(v) Lack of training specific to the preceptor role

Improving preceptorship	Effective collaboration between educational institutions and hospitals
	(i) Improve communication between schools and hospitals
	(ii) Preceptors should account for their stewardship through periodic reports to the schools
	(iii) Regular meetings of stakeholders
	(iv) Schools should select their own preceptors
	(v) Preparation for role
	Improve interdisciplinary approach to teaching
	Financial reward for preceptors
	Streamline preceptorship

## Data Availability

The datasets used and/or analysed during the current study are available from the corresponding author on reasonable request.

## Additional Points

*Limitations of the Study.* A clear weakness in this study, however, is the lack of student voice. Therefore, the results should be interpreted with caution
